# The Jagged-2/Notch-1/Hes-1 Pathway Is Involved in Intestinal Epithelium Regeneration after Intestinal Ischemia-Reperfusion Injury

**DOI:** 10.1371/journal.pone.0076274

**Published:** 2013-10-03

**Authors:** Guoqing Chen, Yuan Qiu, Lihua Sun, Min Yu, Wensheng Wang, Weidong Xiao, Yang Yang, Yong Liu, Songwei Yang, Daniel H. Teitelbaum, Yuanhang Ma, Dingsong Lu, Hua Yang

**Affiliations:** 1 Department of General Surgery, Xinqiao Hospital, Third Military Medical University, Chongqing, China; 2 Department of Surgery, the University of Michigan Medical School, Ann Arbor, Michigan; Charité, Campus Benjamin Franklin, Germany

## Abstract

**Background:**

Notch signaling plays a critical role in the maintenance of intestinal crypt epithelial cell proliferation. The aim of this study was to investigate the role of Notch signaling in the proliferation and regeneration of intestinal epithelium after intestinal ischemia reperfusion (I/R) injury.

**Methods:**

Male Sprague-Dawley rats were subjected to sham operation or I/R by occlusion of the superior mesenteric artery (SMA) for 20 min. Intestinal tissue samples were collected at 0, 1, 2, 4, and 6 h after reperfusion. Proliferation of the intestinal epithelium was evaluated by immunohistochemical staining of proliferating nuclear antigen (PCNA). The mRNA and protein expression levels of Notch signaling components were examined using Real-time PCR and Western blot analyses. Immunofluorescence was also performed to detect the expression and location of Jagged-2, cleaved Notch-1, and Hes-1 in the intestine. Finally, the γ-secretase inhibitor DAPT and the siRNA for Jagged-2 and Hes-1 were applied to investigate the functional role of Notch signaling in the proliferation of intestinal epithelial cells in an *in vitro* IEC-6 culture system.

**Results:**

I/R injury caused increased intestinal crypt epithelial cell proliferation and increased mRNA and protein expression of Jagged-2, Notch-1, and Hes-1. The immunofluorescence results further confirmed increased protein expression of Jagged-2, cleaved Notch-1, and Hes-1 in the intestinal crypts. The inhibition of Notch signaling with DAPT and the suppression of Jagged-2 and Hes-1 expression using siRNA both significantly inhibited the proliferation of IEC-6 cells.

**Conclusion:**

The Jagged-2/Notch-1/Hes-1 signaling pathway is involved in intestinal epithelium regeneration early after I/R injury by increasing crypt epithelial cell proliferation.

## Introduction

Intestinal epithelium covers the surface of the intestine to protect it from various environmental stimuli, including physical and chemical insults and microbial invasion. The intestinal epithelium is one of the most rapidly proliferating tissues in the body [1]. Additionally, among the viscera, the small intestine is most likely the most sensitive and vulnerable to ischemia-reperfusion (I/R) injury [2]. Once the intestinal epithelium is damaged, it activates regeneration programs to restore its continuity and integrated structure through a marked expansion of proliferating undifferentiated progenitor cells [3]. Several signaling pathways and growth factors, such as HB-EGF and KGF, have been reported to be involved in the proliferation of intestinal epithelial cells after I/R injury [4], [5]; however, the precise molecular mechanisms of this process are still not fully understood.

Studies have revealed that the Notch signaling pathway plays critical roles in the maintenance of the intestinal epithelium [6], [7]. Mutations in the Notch receptors are associated with the loss of proliferating progenitor epithelial cells [8]. Notch signaling is an ancient signaling system that plays important roles in cell fate decision and stem cell maintenance in embryonic and postnatal tissues [6], [9]. In mammals, there are four transmembrane Notch receptors, Notch-1, Notch-2, Notch-3, and Notch-4, and five ligands for the receptors, Jagged-1 and Jagged-2, belonging to the serrate family, and Delta-1, Delta-3, and Delta-4, belonging to the Delta family [7]. The interaction of these five ligands with the Notch receptors activates the proteolytic cleavage of the Notch receptors at two distinct sites. This cleavage releases the Notch intracellular domain (NICD), which translocates into the nucleus and functions as a transcriptional activator. Importantly, the second Notch receptor cleavage is mediated by the γ-secretase complex, and the inhibition of this proteolytic activity blocks the activation of Notch receptors [10]. Within the nucleus, NICD forms a large transcriptional activator complex with CSL (RBP-Jk/CBF1) and Mastermind. The transcriptional complex then activates the transcription of target genes, such as Hes (Hairy/Enhancer of split) and Hey (Hes-related with YRPW motif), two families of basic helix-loop-helix genes [11], [12]. Studies have shown that the Notch/Hes-1 signaling pathway controls the proliferation of intestinal immature progenitor cells [13]–[15].

Notch-1 and its ligand Jagged-1 have been shown to promote liver regeneration after partial hepatectomy [16]. Notch signaling is also involved in the regeneration of skin, kidney, heart, pancreas, and tracheal epithelium after injury [17]–[21]. Hes-1 is involved in the adaptation of adult human β-cells that allows them to proliferate in vitro [22]. In the intestinal mucosa of colitis, Watanabe et al. reported that Notch-1/Hes-1 signaling is required for regeneration of intestinal epithelium by increasing the proliferation of intestinal epithelial cells [23]. However, little is known about the role of Notch signaling in the regeneration of intestinal epithelium after I/R injury.

The purpose of the present study was to investigate the relationship between Notch signaling pathway and the regeneration of intestinal epithelium early after I/R injury.

## Materials and Methods

### Animal Experiments

Male Sprague-Dawley (SD) rats weighing 200–250 g were obtained from the Experiment Animal Center at the Daping Hospital of Third Military Medical University. All the animal experiments were performed in compliance with the university’s Guidelines for the Care and Use of Laboratory Animals. The protocol was approved by the ethics committee of Xinqiao Hospital, Third Military Medical University. All surgeries were performed under sodium pentobarbital anesthesia, and all efforts were made to minimize the suffering of the rats. Rats were randomly divided into two groups: control group (sham operation, n = 7) and experimental group (I/R, n = 35). For the I/R rat group, the superior mesenteric artery (SMA) was occluded using an atraumatic microvascular clamp for 20 min [5]. Then, the clamps were removed, the incisions were closed, and seven I/R rats were sacrificed at 0, 1, 2, 4, and 6 h after reperfusion. The rats’ jejunums were quickly removed and processed for histological evaluation, RNA extraction, or protein extraction [24]. The control rats received identical operation procedures without occlusion of the SMA.

### Detection of Epithelial Proliferation

Tissues were fixed with 4% paraformaldehyde, and 5 µm paraffin-embedded sections were prepared. Immunostaining for PNCA was carried out as previously described [24]. The sections were treated with 0.3% hydrogen peroxide in methanol for 20 min. Then, the sections received antigen retrieval by microwave heating for 20 min in citrate buffer (pH 6.0). After blocking with 5% BSA for 20 min, the sections were incubated with a PNCA primary antibody (sc-56, Santa Cruz, CA, USA) overnight at 4°C. The sections were incubated with biotinylated secondary antibody for 20 min at 37°C and avidin-biotin complex for 20 min at 37°C. The peroxidase activities were detected with diaminobenzidine (DAB). After counterstaining with hematoxylin, histological examination was performed under a light microscope.

The crypt cell proliferation rate was calculated as the ratio of the number of crypt cells positive for PCNA staining to the total number of crypt cells. The number of proliferating cells per crypt was defined as the mean of proliferating cells in 10 crypts.

### Immunofluorescence Analysis

For immunofluorescence analysis, 10 µm frozen sections of the jejunum were prepared as previously described [25]. After being fixed in 4% formaldehyde for 20 min, the sections were incubated in 3% H_2_O_2_ for 30 min. Then, the sections were blocked with PBS containing 5% BSA for 30 min at room temperature. The sections were incubated overnight at 4°C with the following primary antibodies diluted in 3% BSA in PBS: Jagged-2 (sc-5604, Santa Cruz, CA, USA), NICD-1 (ab52301, Abcam, UK), and Hes-1 (sc-166410, Santa Cruz, CA, USA). The sections were incubated at 37°C for 60 min with the appropriate secondary antibodies. DAPI (Sigma-Aldrich, St. Louis, MO) was used to stain the nucleus. Images were analyzed and collected with a Leica TCS SP confocal imaging system (Leica, Heidelberg, Germany).

### RT-PCR and Real-Time PCR Analysis

Total RNA was extracted following a standard isothiocyanate/chloroform extraction method using Trizol (Takara Co., Ltd, Dalian, China), and reverse transcription into first-strand cDNA was performed using the First Strand cDNA Synthesis Kit (FSK-100, TOYOBO CO., Ltd, Japan) in the presence of the RNase inhibitor diethylpyrocarbonate (DEPC) (Roche, Germany) [24]. The amplified cDNA was used as the template DNA for PCR performed with TaqDNA polymerase and specific primers. The following PCR primers were used: Jagged-2 forward, 5′- GCGCCAACTGCCACATCAA-3′ and reverse, 5′- GGCTGCTGGCACACTTGTAG-3′; Notch-1 forward, 5′-TGGCCTCAATGGATACAAATG-3′ and reverse, 5′-GGGCCAACACCACCTCAC-3′; and Hes-1 forward, 5′-GGGCAAGAATAAATGAAAG-3′ and reverse, 5′-GCGCGGTACTTCCCCAACAC-3′; GAPDH forward, 5′- GGGGCCAAAAGGGTCATCATCTC-3′ and reverse, 5′- AGGGGCCATCCACAGTCTTC-3′. The RT-PCR steps were as follows: 94°C for 5 min, followed by 34 cycles of 94°C for 30 s, 58°C for 30 s and 72°C for 1 min, and, lastly, 72°C for 5 min. A C1000 Thermal Cycler (Bio-Rad, Singapore) was used. The Kodak Gellogic 212 imaging system (Kodak Molecular Imaging Software, version 5.0; Carestream Health; Rochester, NY, USA) was used for imaging and quantification. The results are expressed as the ratio of the examined mRNA over GAPDH mRNA expression. Real-time PCR was performed as previously described [5]. The standard conditions used for real-time PCR were as follows: 94°C for 10 minutes, 30 seconds at 94°C, 30 seconds at 60°C, and 45 seconds at 72°C for 45 cycles.

### Western Blot Analysis

Tissues and cells were lysed in cold RIPA buffer (PBS, 1% NP-40, 0.5% sodium deoxycholate, 0.1% SDS, 1 µg/ml PMSF, 1.0 mM sodium orthovanadate, and 1×mammalian protease inhibitor cocktail; Sigma-Aldrich). Protein was quantified by the Bradford method using the BCA assay reagent (Beyotime, Shanghai, China). Equal amounts of protein were loaded into SDS–polyacrylamide gels and transferred onto PVDF-Plus membranes. After blocking in 5% skim milk for 1 hour at room temperature, the membranes were incubated overnight at 4°C with the following primary antibodies: Jagged-2 (sc-34476, Santa Cruz, CA, USA), NICD-1 (ab-52301, Abcam, UK), Hes-1 (sc-13844, Santa Cruz, CA, USA) and GAPDH (sc-32233, SantaCruz, CA, USA). Then, the membranes were incubated with horseradish peroxidase-conjugated secondary antibody for 1 h at room temperature and detected by the use of a chemiluminescence system (Beyotime,Shanghai, China) and imaging system (KodakGelLogic 4000 R Imaging System, Carestream, USA). Quantification of the Western blot data were performed by measuring the intensity of the hybridization signals using the Kodak Gel Logic 4000 R Imaging System. Protein expression was normalized to each sample’s expression of GAPDH.

### Cell and Cell Culture

The intestinal epithelial cell line IEC-6, originally purchased from the American type culture collection (ATCC, Manassas, VA), were grown in Dulbecco’s modified Eagles medium (DMEM, Hyclone, Thermo Fisher; Rockford, IL) supplemented with 10% fetal calf serum (Sigma-Aldrich; St. Louis, MO), 100 µg/ml streptomycin and 100 IU/ml penicillin and cultured overnight for adhesion. Once grown, the IEC-6 cells were cultured at 37°C in either normoxic (20% O_2_ and 5% CO_2_) or hypoxic (1% O_2_ and 5% CO_2_ in a hypoxia chamber) conditions (Thermo Fisher Scientific, Ohio, USA). The γ-secretase inhibitor DAPT was added to the medium for 48 h, and the cell count and proliferation of IEC-6 cells were investigated. Total protein was also obtained for Western blot analysis.

For the MTT assay, IEC-6 cells were seeded in 96-well plates and incubated in fresh medium with DMSO or the γ-secretase inhibitor DAPT. Cell proliferation was detected by the MTT assay (Boster, Wuhan, China). After culture for 48 h, the supernatant was removed and 20 µl of MTT reagent were added to each well of a 96-well plate, and the plates were incubated for 4 h. Then, the supernatant was removed, and the cells were treated with 100 µl/well formazan solution for 10 min. Absorbance at 570 nm was recorded using an ELISA plate reader.

### Silencing Jagged-2 and Hes-1 Using siRNA and In Vitro Transfection

To inhibit the Notch signaling pathway, an *in vitro* transfection to silence Jagged-2 and Hes-1 expression was performed. siRNA were chemically synthesized by Ribobio (Guangzhou, China). The sequences of the siRNAs targeting Jagged-2 were as follows: siRNA1, sense 5′ GCAAAGAAGCCGUGUGUAA dTdT3′ and antisense 3′ dTdT CGUUUCUUCGGCACACAUU5′; siRNA2, sense 5′ CCACUCAUUUGGACAACAA dTdT 3′ and antisense 3′ dTdT GGUGAGUAAACCUGUUGUU 5′; The sequences of the siRNAs targeting Hes-1 were as follows: siRNA1, sense 5′ GAAGGCAGACAUUCUGGAA dTdT 3′ and antisense 3′ dTdT CUUCCGUCUGUAAGACCUU 5′; siRNA2, sense 5′ GGAUGCACUUAAGAAAGAU dTdT 3′ and antisense 3′ dTdT CCUACGUGAAUUCUUUCUA 5′.After IEC-6 cells were cultured to 50–60% confluency in 6-well plates, they were transfected with siRNA at a concentration of 50 nmol for each well using the Lipofectamine 2000 reagent (Invitrogen) in antibiotic-free and serum-free Opti-MEM medium, according to the manufacturer’s instructions. An unrelated control siRNA (si-NC) was used in the experiment as the negative control. After 6 h, the medium was replaced with normal IEC-6 cell medium, and the cells were cultured for 48 h. Then, cell counts were performed. Protein was extracted from the cells, and Western blot analysis was performed to detect the protein expression of Jagged-2, NICD-1, and Hes-1.

To investigate the effect of siRNA on the proliferation of IEC-6 cells, cells were plated onto a 96-well plate, and suppression of Jagged-2 and Hes-1 using siRNA was carried out according to the manufacturer’s instructions, as mentioned above. The MTT assay was applied to examine the cell number of IEC-6 cells.

### Statistical Analysis

All the results are presented as the mean ± SD. The Student’s t test was used for the comparisons of the mean values between two groups. ANOVA was used for comparisons of more than two groups. All statistical analyses were carried out using SPSS13.0 software. *P*<0.05 was considered significant.

## Results

### Intestinal Epithelial Proliferation Increased After I/R

**Figure 1:**
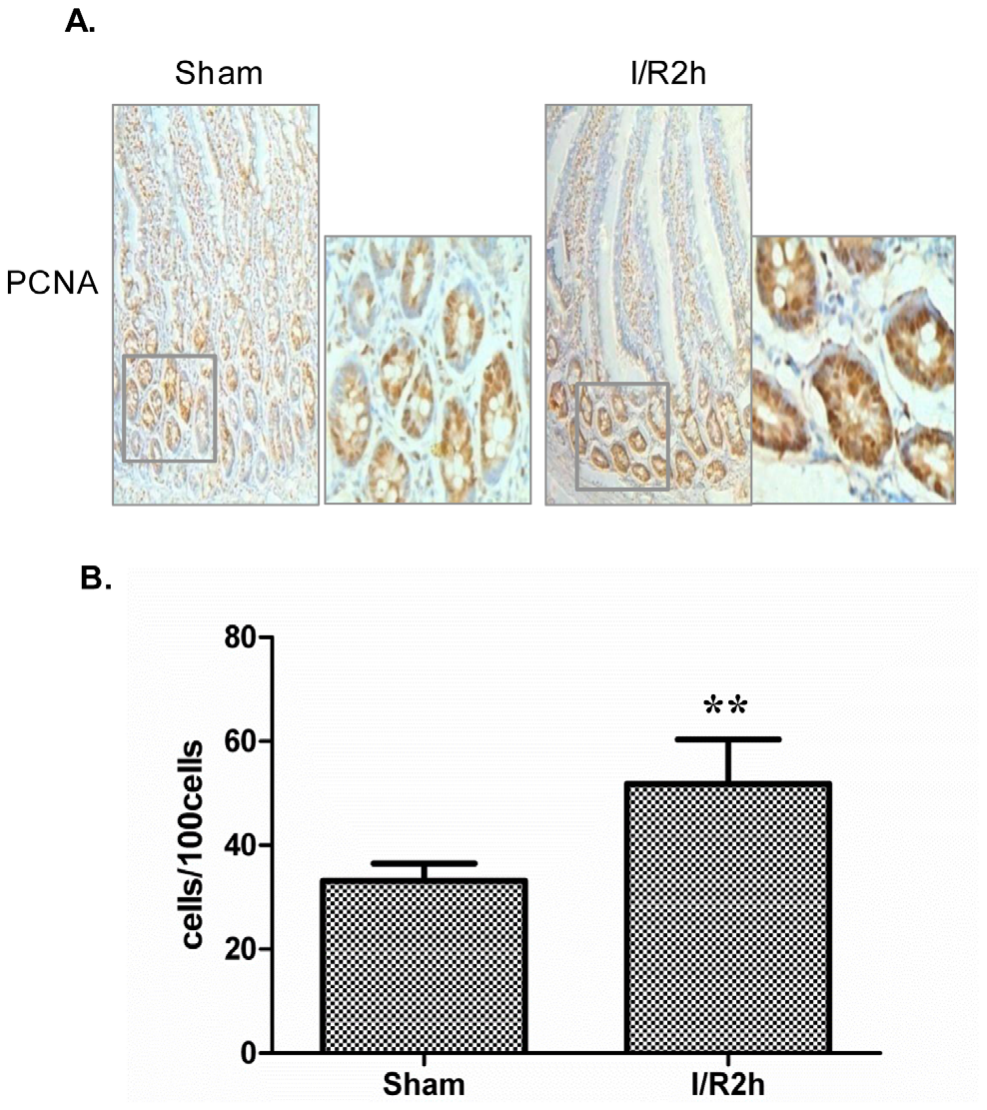
PCNA expression is increased in intestinal crypt epithelial cells after I/R (2 h). *A*: Immunostaining of rat intestinal tissues showing expression of PCNA. Brown staining showed positive results for PCNA (original magnification ×200). Magnified view of the squared area is shown in the right of the original picture (original magnification ×400). *B*: Graphic representation of relative number of PCNA positive cells. Data are given as the means ± SDs (*n* = 7). **p<0.01 versus sham group.

Jejunum of the rats were excised at 2 h after I/R. Immunohistochemical results showed that the PCNA-positive cells were isolated to the crypts of the small intestine. Importantly, I/R (2 h) significantly increased the number of PCNA positive cells compared with sham operation (Fig. 1a, b). The PCNA positive cells increased 1.56-fold in the I/R (2 h) group compared with the sham group (Fig. 1b). There was no difference in the location of PCNA positive cells between the groups, and all the counted PCNA positive cells were found in the crypts.

### The Notch Signaling Cascade was Activated in a Rat Model of I/R

**Figure 2:**
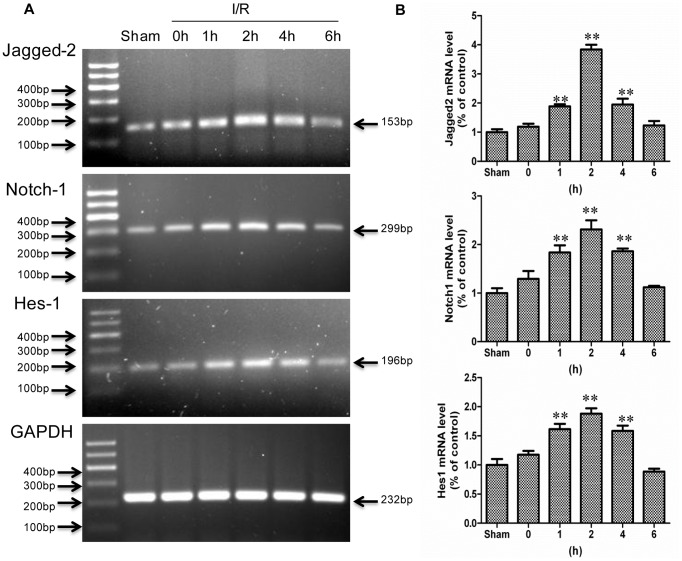
Transcripts of Notch signaling components were increased in intestine after I/R injury. *A*: The I/R and sham-operated rats were sacrificed at indicated times, mRNA of intestinal mucosa was collected, and RT-PCR was performed to detect mRNA levels of Jagged-2, Notch-1, and Hes-1. GAPDH was used to verify equivalent loading. *B*: mRNA expression of Jagged-2, Notch-1, and Hes-1 detected by Real-time PCR. Data are given as the means ± SDs (*n* = 7). **p<0.01 versus sham group.

To investigate the expression of Notch signaling components in intestinal epithelium after I/R, we generated a rat model of I/R by occlusion of the SMA for 20 min. The clamps were removed, and I/R rats were killed at 0, 1, 2, 4, and 6 h after the initial reperfusion. As shown in Fig. 2, A and B, the mRNA levels of Jagged-2, Notch-1, and the target gene Hes-1 were weakly detected in the intestine of sham operated rats compared to the I/R rats and gradually increased in the I/R rats. The mRNA expression of Jagged-2, Notch-1, and Hes-1 increased 3.84, 2.31 and 1.88-fold at 2 h after I/R compared to sham operation, respectively (Fig. 2b, p<0.01 I/R 2 h vs. Sham) and gradually returned to normal levels at 6 h after I/R. Based on the mRNA expression of Notch signaling components, we focused on Jagged-2, Notch-1, and Hes-1 during the following experiments.

**Figure 3:**
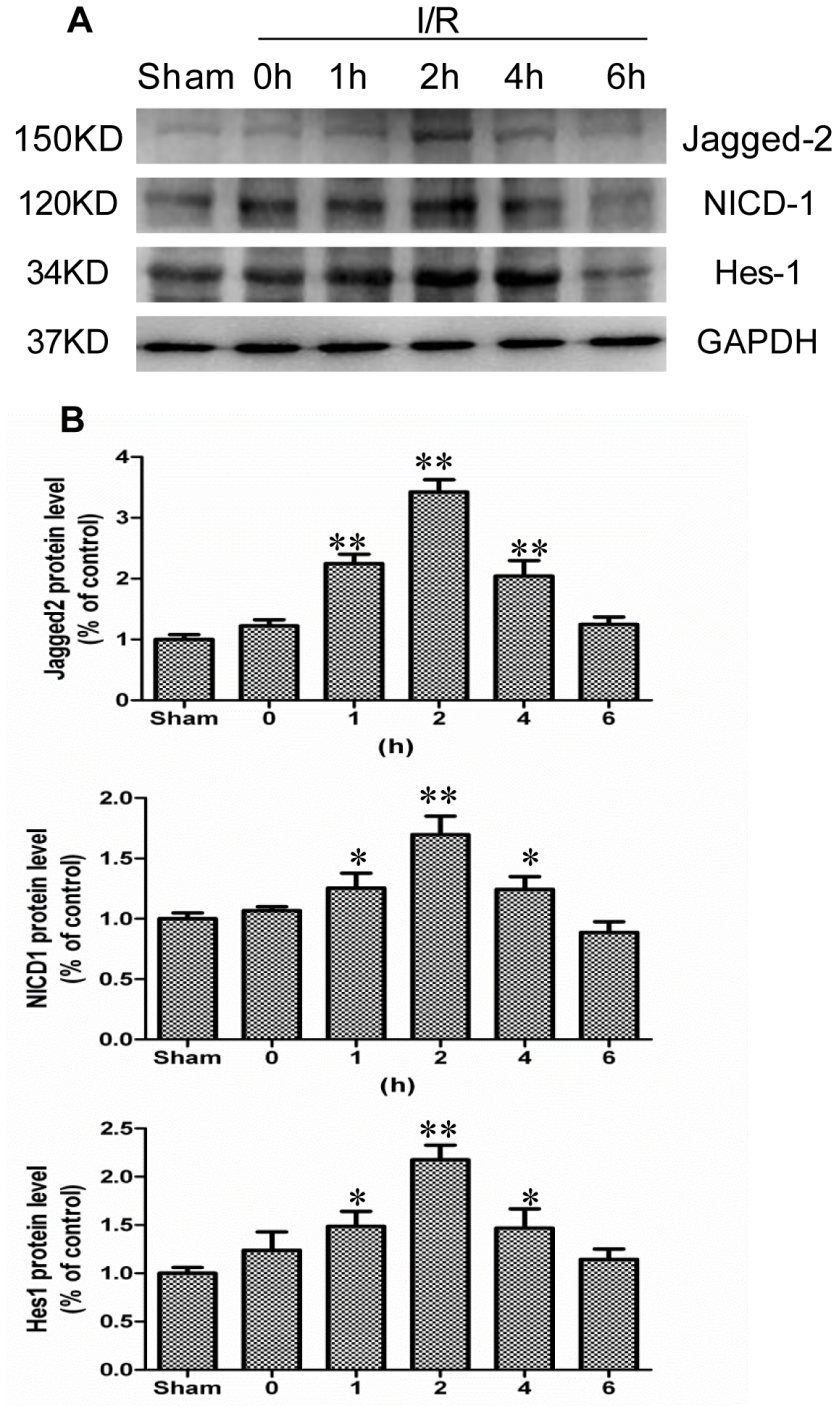
The protein expressions of Notch signaling components were increased in intestine after I/R injury. *A*: The I/R and sham-operated rats were sacrificed at indicated times, and Western blot was performed to detect protein expression of Jagged-2, NICD-1, and Hes-1. GAPDH was used to verify equivalent loading. *B*: Graphic representation of relative expression of Jagged-2, NICD-1, and Hes-1 normalized to GAPDH. Data are given as the means ± SDs (*n* = 7). **p<0.01 versus sham group. *p<0.05 versus sham group.

The protein levels of Jagged-2 and Hes-1 also increased gradually after I/R injury (Fig. 3, a, b). The protein expressions of Jagged-2 and Hes-1 were increased 3.42 and 2.18-fold after I/R injury, respectively (p<0.01 I/R2h vs. Sham). Because cleavage of Notch-1 is the indicator of Notch signal activation, we investigated NICD-1 expression using an antibody specific to the fragment of activated Notch-1. Western blot analyses showed a significant increase in the NICD-1 level at 2 h after I/R, indicating the activation of Notch signaling (Fig. 3a). Quantification of the Western blot results revealed a 1.72-fold increase of NICD-1 compared with the sham-operated rats (p<0.01 I/R 2 h vs. Sham) (Fig. 3b).

**Figure 4:**
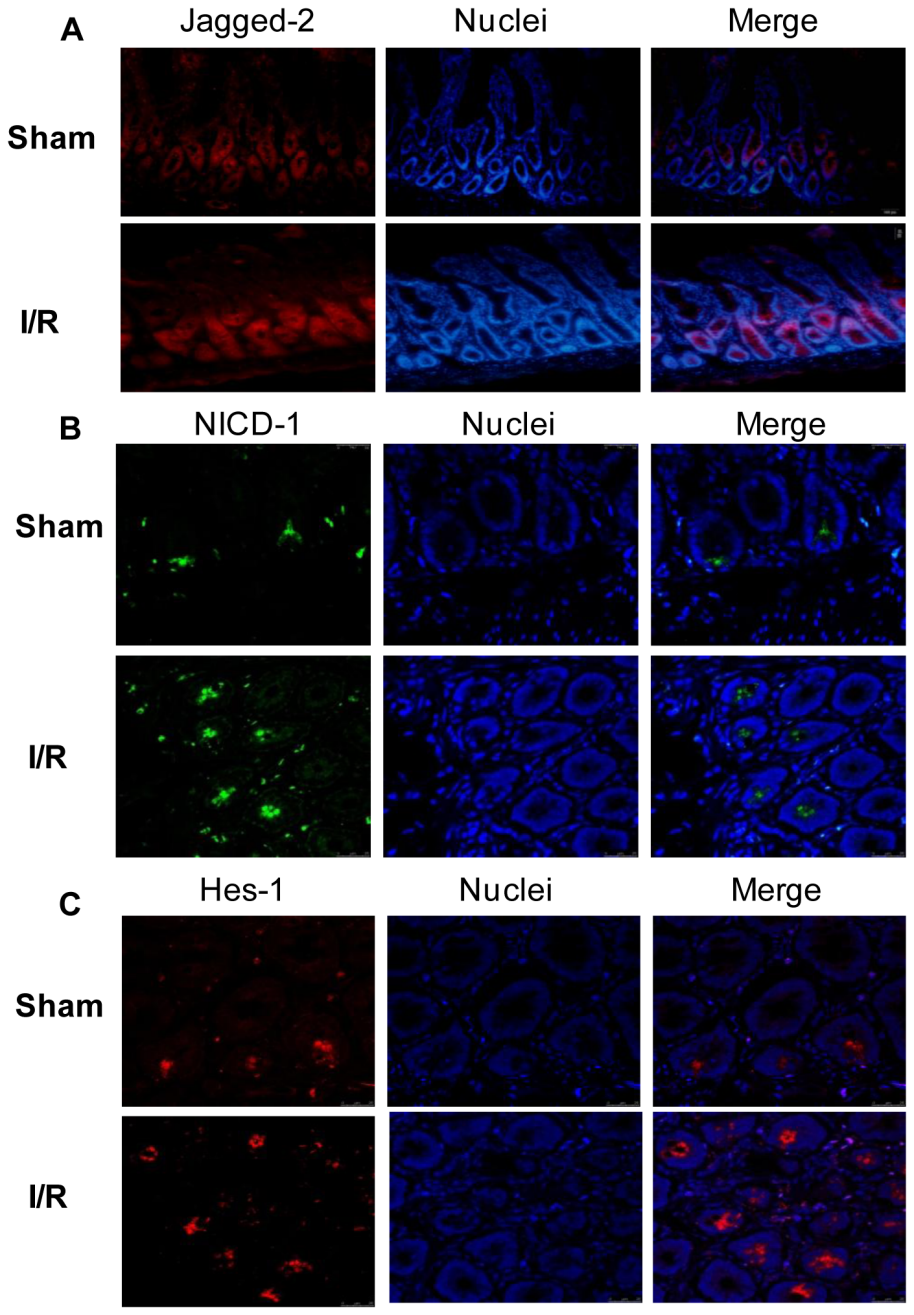
Immunofluorescence evidence for increased Notch signaling activation in intestine after I/R injury. Frozen sections of intestine from sham operated rats or rats after I/R injury were stained with antibodies against Jagged-2 (A), NICD-1 (B), and Hes-1 (C). Nuclei were stained with DAPI (blue). Images (A: magnification×200; B, C: magnification×400) were taken by confocal microscopy.

The protein expression of Jagged-2, NICD-1, and Hes-1 was further investigated by immunofluorescence. As shown in Fig. 4, A–C, positive staining for Jagged-2, NICD-1, and Hes-1 was readily detected in the intestinal crypt epithelial cells after 2 h of I/R. In contrast, considerably fewer cells with positive staining were detected in the intestine of sham operated rats (Fig. 4, a–c). These data indicated that Notch signaling was upregulated in the proliferating intestinal crypt epithelial cells. Notch signaling was highly activated and may be correlated with the proliferation of crypt epithelial cells after intestinal I/R injury.

### Effects of DAPT on the Proliferation of IEC-6 Cells

**Figure 5:**
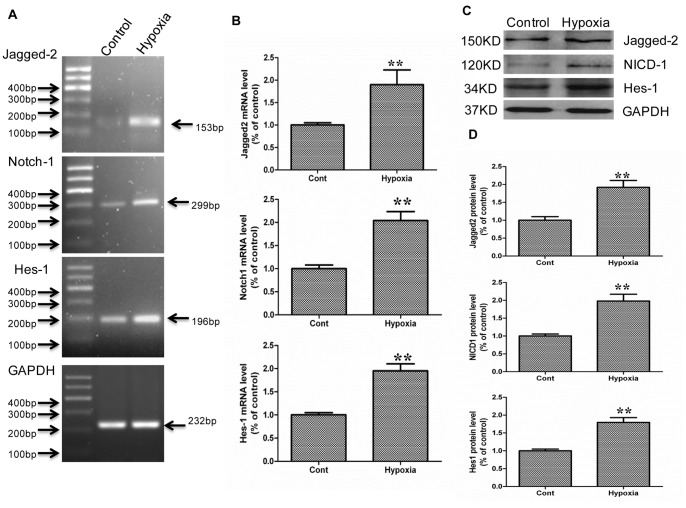
The mRNA and protein expressions of Jagged-2, Notch-1, and Hes-1 for IEC-6 cells under a hypoxia condition. A: The mRNA extracted from IEC-6 cells was used for RT-PCR analysis of Jagged-2, Notch-1, Hes-1, and GAPDH mRNA levels. B: mRNA expression of Jagged-2, Notch-1, and Hes-1 detected by Real-time PCR. Data are shown as the means ± SDs (*n* = 5). **p<0.01 versus control group. C: Protein was extracted from IEC-6 cells. Western blot analysis was performed to detect the protein expression of Jagged-2, NICD-1, and Hes-1. GAPDH was used as the loading control. D: Quantitative analyses of Western blot results were performed for Jagged-2, NICD-1, and Hes-1. Data are shown as the means ± SDs (*n* = 5). **p<0.01 versus control group.

Next, we used a culture system with IEC-6 cells to examine the functional role of the Jagged-2/NICD-1/Hes-1 signaling pathway in intestinal epithelial cells. First, to examine whether this culture system mimics the *in vivo* ischemia/reperfusion model, we investigated the mRNA expression of Jagged-2, Notch-1, and Hes-1 and the protein expression of Jagged-2, NICD-1, and Hes-1 by Western blot. The PCR results showed that hypoxia (6 h) upregulated the mRNA expression of Jagged-2, Notch-1, and Hes-1 by 1.88, 2.04, and 1.95-fold compared to the control IEC-6 cells, respectively (p<0.01 hypoxia vs. Sham) (Fig. 5, a, b). In the western blot analysis, hypoxia upregulated the protein expression of Jagged-2, NICD-1, and Hes-1 by 1.9, 1.98, and 1.79-fold compared to the control levels, respectively (p<0.01 hypoxia vs. Sham) (Fig. 5, c, d). These results indicated that the IEC-6 culture system mimics the *in vivo* ischemia/reperfusion model.

**Figure 6:**
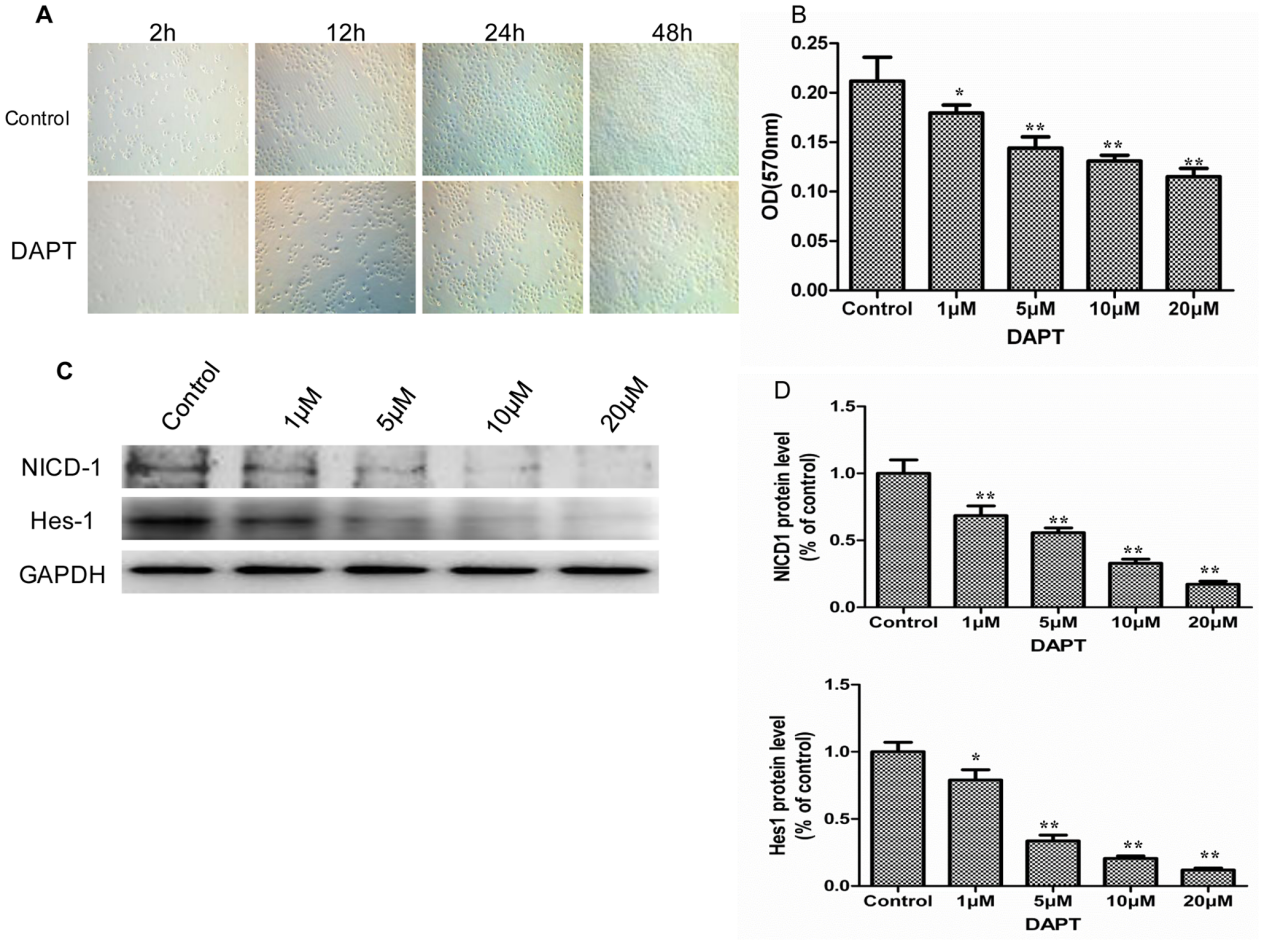
Inhibition of Notch signaling by DAPT suppressed cell proliferation of IEC-6 cells. *A*: IEC-6 cells with DMSO (Control) or DAPT (20 µM) for indicated times (magnification×100). *B*: IEC-6 cells were plated onto a 96-well culture plate, and DAPT was added. MTT assay was performed to examine the proliferation of IEC-6 cells. Data are given as the means ± SDs (*n* = 10). *C*: Protein was extracted from IEC-6 cells plated onto a 6-well plate, and protein expression of NICD-1, and Hes-1 was examined by Western blot. GAPDH was used as the loading control. *D*: Graphic representation of relative expression of NICD-1, and Hes-1 normalized to GAPDH. Data are given as the means ± SDs (*n* = 5). **p<0.01 versus control group. *p<0.05 versus control group.

DAPT was used to stimulate the IEC-6 cells to investigate the function of Notch signaling in intestinal epithelial cells. DAPT is one type of γ-secretase inhibitor that inhibits the activation of Notch signaling. After DAPT was added to the culture medium of IEC-6 cells, the cell morphologies were observed, and cell counts were taken repeatedly over the course of 48 h. The IEC-6 cell count in the presence of DAPT was significantly reduced when compared with the control group (Fig. 6a, b). Furthermore, we examined the effects of DAPT on the proliferation of IEC-6 cells by MTT assay. The result showed that DAPT reduced the IEC-6 cell number dose-dependently (Fig. 6b). These results demonstrated that DAPT inhibited the proliferation of IEC-6 cells and Notch signaling was involved in the proliferation of intestinal epithelial cells. To explore these results further, we examined the effect of DAPT on the signal transduction of IEC-6 cells. The Western blot results showed that DAPT downregulated the protein expression of NICD-1 and Hes-1 dose-dependently (Fig. 6c, d). Taken together, these results confirmed that NICD-1/Hes-1 signaling plays an important role in the proliferation of intestinal crypt epithelial cells.

### Effects of Silencing RNA for Jagged-2 and Hes-1 on the Proliferation of IEC-6 Cells

**Figure 7:**
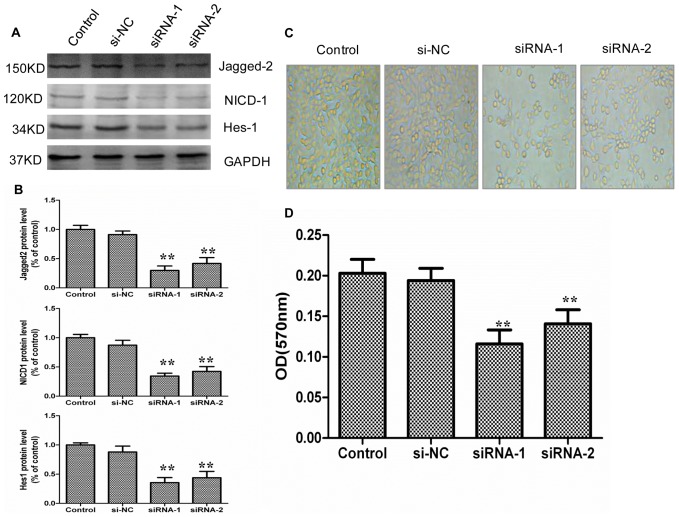
siRNA for Jagged-2 inhibited proliferation of IEC-6 cells. Cells were plated onto 6-well plates. Inhibition of Jagged-2 with siRNA was carried out as mentioned above (see the Materials and Methods). *A*: After 48 h of culture, protein was extracted from IEC-6 cells plated on 6-well plates. Western blot analysis showed that siRNA for Jagged-2 downregulated protein expression of Jagged-2, NICD-1, and Hes-1 of IEC-6 cells compared with the si-NC or control group. GAPDH was used as loading control. *B*: Graphic representation of relative expression of Jagged-2, NICD-1, and Hes-1 normalized to GAPDH. Data are given as the means ± SDs (*n* = 5). **p<0.01 versus control group. *C*: Cell count of IEC-6 cells was taken for control group, si-NC, and siRNA for Jagged-2 group (magnification×400). *D*: IEC-6 cells were plated onto a 96-well plate. Inhibition of Jagged-2 with siRNA was carried out as mentioned above (see the Materials and Methods). MTT assay showed that siRNA for Jagged-2 decreased the cell number of IEC-6 cells significantly. Data are given as the means ± SDs (*n* = 10) **p<0.01 versus control group. Control: IEC-6 cells were cultured normally. si-NC: IEC-6 cells were transfected with unrelated control siRNA. siRNA: IEC-6 cells were transfected with siRNA for Jagged-2.

To further investigate the effect of Jagged-2/NICD-1/Hes-1 signaling on intestinal epithelial cell proliferation, IEC-6 cells were transfected with siRNA for Jagged-2 and Hes-1. The Western blot results showed that the siRNA for Jagged-2 significantly downregulated the protein expression of Jagged-2 (Fig. 7a, b). The protein expressions of NICD-1 and Hes-1 were also downregulated significantly (Fig. 7a, b). These results showed that Notch signaling was inhibited significantly by the siRNA for Jagged-2. At the same time, suppression of Jagged-2 expression decreased the IEC-6 cell count compared with the control group (Fig. 7c, d). We also examined the effect of the siRNA for Jagged-2 on the proliferation of IEC-6 cells by MTT assay. The MTT result showed that the siRNA for Jagged-2 significantly reduced the IEC-6 cell number (Fig. 7d).

**Figure 8:**
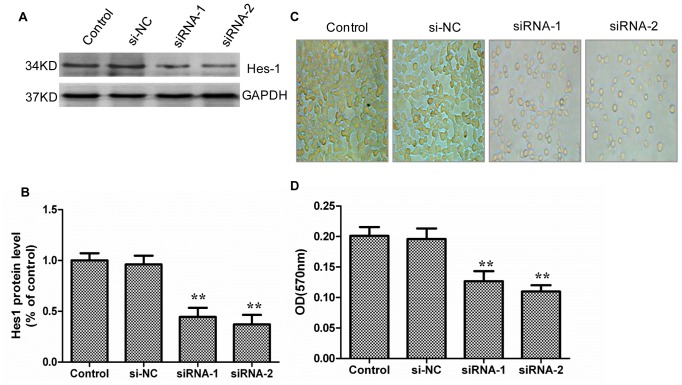
siRNA for Hes-1 inhibited proliferation of IEC-6 cells. Cells were plated onto 6-well plates. Inhibition of Jagged-2 with siRNA was carried out as mentioned above (see the Materials and Methods). *A*: After 48 h of culture, protein was extracted from IEC-6 cells. Western blot analysis showed that siRNA for Hes-1 downregulated protein expression of Hes-1 in IEC-6 cells compared with the si-NC or control group. GAPDH was used as loading control. *B*: Graphic representation of relative expression of Hes-1 normalized to GAPDH. Data are given as the means ± SDs (*n* = 5). **p<0.01 versus control group. *C*: Cell count of IEC-6 cells was taken for control group, si-NC, and siRNA for Hes-1 group (magnification×400). *D*: IEC-6 cells were plated onto a 96-well plate. Inhibition of Hes-1 with siRNA was carried out as mentioned above (see the Materials and Methods). MTT assay showed that siRNA for Hes-1 decreased the cell number of IEC-6 cells significantly. Data are given as the means ± SDs (*n* = 10) **p<0.01 versus control group. Control: IEC-6 cells were cultured normally. si-NC: IEC-6 cells were transfected with unrelated control siRNA. siRNA: IEC-6 cells were transfected with siRNA for Hes-1.

In addition, the Western blot results showed that the siRNA for Hes-1 also significantly downregulated the protein expression of Hes-1 (Fig. 8a, b). At the same time, suppression of Hes-1 expression decreased the IEC-6 cell count compared with the control group (Fig. 8c, d). We also examined the effect of the siRNA for Hes-1 on the proliferation of IEC-6 cells by MTT assay. The MTT result showed that the siRNA for Hes-1 significantly reduced the IEC-6 cell number (Fig. 8d).

Taken together, these results confirmed that the siRNA for Jagged-2 and Hes-1 inhibits the proliferation of IEC-6 cells and that the Jagged-2/NICD-1/Hes-1 signaling pathway is involved in the proliferation of intestinal crypt epithelial cells.

## Discussion

In this study, we found that intestinal I/R stress caused a transient rapid proliferation of intestinal crypt epithelial cells early after reperfusion. The mRNA and protein expression of Jagged-2, Notch-1, and Hes-1 were significantly increased in intestinal crypt epithelial cells early after I/R injury. Our findings also demonstrated that the Jagged-2/Notch-1/Hes-1 signaling pathway is involved in the proliferation of intestinal crypt epithelial cells.

Intestinal I/R injury, which is associated with hemorrhage, ischemia and other shock states, is characterized by alterations in capillary permeability, mucosal barrier dysfunction, necrosis and epithelial shedding. Mucosal damage is followed by transient rapid proliferation of intestinal crypt epithelial cells, migration of new cells from the crypt to the villus, and subsequent differentiation of villous cells to rebuild the proper structure of the epithelium [26], [27]. Studies have shown that the proliferation of intestinal epithelial cells occurs in the early phase after I/R, and immediate early genes, such as c*-fos* and c*-jun,* are involved in the proliferation of intestinal epithelial cells [28], [29]. Some other factors, such as histamine, histidine decarboxylase, and ornithine decarboxylase, were also reported to be involved in the regeneration of intestinal mucosa after I/R [30], [31]. However, the precise molecular mechanisms that accompany I/R within the intestinal epithelium remain to be elucidated.

The Notch signaling pathway plays critical roles in the maintenance of proliferating crypt epithelial cells [6]. Mutations in the Notch pathway components, such as the Notch DNA-binding protein RBP-J or Notch receptors, lead to decreased proliferation of intestinal crypt epithelial cells [8], [32]–[34]. Inhibition of Notch signaling activation through γ-secretase inhibitors also decreases the proliferation of intestinal crypt epithelial cells [32]–[34]. Conversely, the activation of Notch signaling significantly increases the proliferation of intestinal epithelial cells [13], [14]. All these studies suggest that Notch signaling plays indispensable roles in the regulation of intestinal proliferating crypt epithelial cells. In this study, we further confirmed the expression and function of Notch signaling in the regeneration of the intestinal epithelium after I/R.

To our knowledge, this study is the first to demonstrate the upregulation of Jagged-2 expression in the early phase after intestinal I/R. Physiologically, Jagged-2 expression is localized mainly in the intestinal crypts, suggesting its role in the regulation of crypt epithelial cell proliferation [35], [36]. Other studies have shown that Jagged-2 expression was strongly upregulated by hypoxia in breast tumor cells and bone marrow stoma and that this upregulation promoted the growth of cancer stem-like cells through the activation of Notch signaling [37]. Jagged-2 mediated Notch signaling also played critical roles in the proliferation of neural progenitors and hematopoietic progenitors [38], [39]. In this study, we detected increased Jagged-2 expression in the intestine after I/R. Two hours after I/R, Jagged-2 protein expression increased significantly and gradually returned to its normal level at 6 h after I/R. Corresponding with the Jagged-2 expression increase, the protein expression of NICD-1 and Hes-1 was also upregulated significantly at 2 h after I/R. To further confirm the activation of Notch signaling after I/R, immunofluorescence examination of the intestinal tissues was carried out. The results showed that the expression of Notch signaling components was located in the intestinal crypts and that the number of cells that expressed Notch signaling components increased significantly after I/R. These results showed that Notch signaling was activated in the intestine after I/R and may be involved in the proliferation of crypt epithelial cells after I/R.

In addition, an IEC-6 cell culture system was applied to investigate the functional role of Notch signaling in intestinal crypt epithelial cell proliferation. In some instances, it may be inappropriate to extend *in vitro* results to *in vivo* conditions; thus, we performed Real-time PCR and Western blot analysis to examine whether hypoxia activates Jagged-2, Notch-1, and Hes-1 in IEC-6 cells. The results showed that the mRNA and protein expression of Jagged-2, cleaved Notch-1, and Hes-1 was upregulated in IEC-6 cells under the hypoxia condition. These results suggested that the IEC-6 cells could mimic the in vivo I/R model upon induction of the Jagged-2/Notch-1/Hes-1 signaling pathway. Next, the γ-secretase inhibitor DAPT, which inhibits γ-secretase activation of Notch receptors, was used to clarify the function of Notch signaling on the proliferation of IEC-6 cells. The results showed that the protein expressions of NICD-1 and Hes-1 were downregulated significantly. The IEC-6 cell count decreased and that the proliferation of IEC-6 cells was inhibited significantly. Next, siRNA for Jagged-2 was used to suppress the expression of Jagged-2 and examine the role of Jagged-2-Notch signaling in the proliferation of IEC-6 cells. The results showed that suppression of Jagged-2 with siRNA downregulated Notch signaling and significantly inhibited the proliferation of IEC-6 cells. Finally, siRNA for Hes-1 was applied to examine the relationship between Hes-1 expression and proliferation of IEC-6 cells. The results showed that Hes-1 expression was involved in the proliferation of IEC-6 cells.

All these results suggest that the Jagged-2/Notch-1/Hes-1 signaling pathway is involved in the proliferation of intestinal crypt epithelial cells. In addition to the Notch signaling pathway, the Hedgehog, Wnt, and Bmps pathways also play important roles in the homeostasis of the intestinal epithelium and in the establishment of the Crypt-Villus Axis [40]. The roles of these factors and their interactions with the Notch signaling pathway during the regeneration of intestinal epithelium after I/R injury require further investigation.

In summary, our study showed that the Jagged-2/Notch-1/Hes-1 signaling pathway was activated and involved in intestinal crypt epithelial cell proliferation after I/R. The Jagged-2/Notch-1/He-1 signaling pathway was involved in the regeneration of the intestinal epithelium early after I/R. Further studies are necessary to gain a more precise understanding of the molecular mechanisms of intestinal regeneration after I/R.

## References

[1] Johnson LR (1988) Regulation of gastrointestinal mucosal growth. Physiol Rev 68: 456–502. 3282244 10.1152/physrev.1988.68.2.456

[2] Yamamoto S, Tanabe M, Wakabayashi G, Shimazu M, Matsumoto K, et al (2001) The role of tumor necrosis factor-alpha and interleukin-1beta in ischemia-reperfusion injury of the rat small intestine. J Surg Res 99: 134–141. 11421615 10.1006/jsre.2001.6106

[3] Okamoto R, Watanabe M (2004) Molecular and clinical basis for the regeneration of human gastrointestinal epithelia. J Gastroenterol 39: 1–6. 14767727 10.1007/s00535-003-1259-8

[4] El-Assal ON, Besner GE (2004) Heparin-binding epidermal growth factor-like growth factor and intestinal ischemia-reperfusion injury. Semin Pediatr Surg 13: 2–10. 14765365 10.1053/j.sempedsurg.2003.09.002

[5] Cai Y, Wang W, Liang H, Sun L, Teitelbaum DH, et al (2012) Keratinocyte growth factor improves epithelial structure and function in a mouse model of intestinal ischemia/reperfusion. PLoS One 7: e44772. 23028616 10.1371/journal.pone.0044772 PMC3441439

[6] Artavanis-Tsakonas S, Rand MD, Lake RJ (1999) Notch signaling: cell fate control and signal integration in development. Science 284: 770–776. 10221902 10.1126/science.284.5415.770

[7] Baron M (2003) An overview of the Notch signalling pathway. Semin Cell Dev Biol 14: 113–119. 12651094 10.1016/s1084-9521(02)00179-9

[8] Riccio O, van Gijn ME, Bezdek AC, Pellegrinet L, van Es JH, et al (2008) Loss of intestinal crypt progenitor cells owing to inactivation of both Notch1 and Notch2 is accompanied by derepression of CDK inhibitors p27Kip1 and p57Kip2. EMBO Rep 9: 377–383. 18274550 10.1038/embor.2008.7 PMC2288761

[9] Huppert SS, Le A, Schroeter EH, Mumm JS, Saxena MT, et al (2000) Embryonic lethality in mice homozygous for a processing-deficient allele of Notch1. Nature 405: 966–970. 10879540 10.1038/35016111

[10] Artavanis-Tsakonas S, Rand MD, Lake RJ (1999) Notch signaling: cell fate control and signal integration in development. Science 284: 770–776. 10221902 10.1126/science.284.5415.770

[11] Bailey AM, Posakony JW (1995) Suppressor of hairless directly activates transcription of enhancer of split complex genes in response to Notch receptor activity. Genes Dev 9: 2609–2622. 7590239 10.1101/gad.9.21.2609

[12] Kokubo H, Lun Y, Johnson RL (1999) Identification and expression of a novel family of bHLH cDNAs related to Drosophila hairy and enhancer of split. Biochem Biophys Res Commun 260: 459–465. 10403790 10.1006/bbrc.1999.0880

[13] Fre S, Huyghe M, Mourikis P, Robine S, Louvard D, et al (2005) Notch signals control the fate of immature progenitor cells in the intestine. Nature 435: 964–968. 15959516 10.1038/nature03589

[14] Stanger BZ, Datar R, Murtaugh LC, Melton DA (2005) Direct regulation of intestinal fate by Notch. Proc Natl Acad Sci U S A 102: 12443–12448. 16107537 10.1073/pnas.0505690102 PMC1194941

[15] Murata K, Hattori M, Hirai N, Shinozuka Y, Hirata H, et al (2005) Hes1 directly controls cell proliferation through the transcriptional repression of p27Kip1. Mol Cell Biol 25: 4262–4271. 15870295 10.1128/MCB.25.10.4262-4271.2005 PMC1087711

[16] Kohler C, Bell AW, Bowen WC, Monga SP, Fleig W, et al (2004) Expression of Notch-1 and its ligand Jagged-1 in rat liver during liver regeneration. Hepatology 39: 1056–1065. 15057910 10.1002/hep.20156 PMC1769555

[17] Adolphe C, Wainwright B (2005) Pathways to improving skin regeneration. Expert Rev Mol Med 7: 1–14. 10.1017/S1462399405009890 16179092

[18] Kobayashi T, Terada Y, Kuwana H, Tanaka H, Okado T, et al (2008) Expression and function of the Delta-1/Notch-2/Hes-1 pathway during experimental acute kidney injury. Kidney Int 73: 1240–1250. 18418349 10.1038/ki.2008.74

[19] Croquelois A, Domenighetti AA, Nemir M, Lepore M, Rosenblatt-Velin N, et al (2008) Control of the adaptive response of the heart to stress via the Notch1 receptor pathway. J Exp Med 205: 3173–3185. 19064701 10.1084/jem.20081427 PMC2605223

[20] Siveke JT, Lubeseder-Martellato C, Lee M, Mazur PK, Nakhai H, et al (2008) Notch signaling is required for exocrine regeneration after acute pancreatitis. Gastroenterology 134: 544–555. 18242220 10.1053/j.gastro.2007.11.003

[21] Ma XB, Jia XS, Liu YL, Wang LL, Sun SL, et al (2009) Expression and role of Notch signalling in the regeneration of rat tracheal epithelium. Cell Prolif 42: 15–28. 19143760 10.1111/j.1365-2184.2008.00569.x PMC6496151

[22] Bar Y, Russ HA, Knoller S, Ouziel-Yahalom L, Efrat S (2008) HES-1 is involved in adaptation of adult human beta-cells to proliferation in vitro. Diabetes 57: 2413–2420. 18599525 10.2337/db07-1323 PMC2518492

[23] Okamoto R, Tsuchiya K, Nemoto Y, Akiyama J, Nakamura T, et al (2009) Requirement of Notch activation during regeneration of the intestinal epithelia. Am J Physiol Gastrointest Liver Physiol 296: G23–35. 19023031 10.1152/ajpgi.90225.2008

[24] Chen G, Sun L, Yu M, Meng D, Wang W, et al.. (2013) The Jagged-1/Notch-1/Hes-1 Pathway Is Involved in Intestinal Adaptation in a Massive Small Bowel Resection Rat Model. Dig Dis Sci. 10.1007/s10620-013-2680-3 23595520

[25] Wang W, Xiao W, Sun L, Zhang C, Chen G, et al (2012) Inhibition of ACE activity contributes to the intestinal structural compensation in a massive intestinal resection rat model. Pediatr Surg Int 28: 533–541. 22441330 10.1007/s00383-012-3075-9

[26] Rijke RP, Hanson WR, Plaisier HM, Osborne JW (1976) The effect of ischemic villus cell damage on crypt cell proliferation in the small intestine: evidence for a feedback control mechanism. Gastroenterology 71: 786–792. 964570

[27] Yeh KY, Yeh M, Glass J (1998) Expression of intestinal brush-border membrane hydrolases and ferritin after segmental ischemia-reperfusion in rats. Am J Physiol 275: G572–583. 9724271 10.1152/ajpgi.1998.275.3.G572

[28] Shima Y, Tajiri T, Taguchi T, Suita S (2006) Increased expression of c-fos and c-jun in the rat small intestinal epithelium after ischemia-reperfusion injury: a possible correlation with the proliferation or apoptosis of intestinal epithelial cells. J Pediatr Surg 41: 830–836. 16567203 10.1016/j.jpedsurg.2005.12.025

[29] Itoh H, Yagi M, Hasebe K, Fushida S, Tani T, et al (2002) Regeneration of small intestinal mucosa after acute ischemia-reperfusion injury. Dig Dis Sci 47: 2704–2710. 12498289 10.1023/a:1021049004188

[30] Fujimoto K, Imamura I, Granger DN, Wada H, Sakata T, et al (1992) Histamine and histidine decarboxylase are correlated with mucosal repair in rat small intestine after ischemia-reperfusion. J Clin Invest 89: 126–133. 1729265 10.1172/JCI115552 PMC442827

[31] Fujimoto K, Granger DN, Price VH, Tso P (1991) Ornithine decarboxylase is involved in repair of small intestine after ischemia-reperfusion in rats. Am J Physiol 261: G523–529. 1887898 10.1152/ajpgi.1991.261.3.G523

[32] van Es JH, van Gijn ME, Riccio O, van den Born M, Vooijs M, et al (2005) Notch/gamma-secretase inhibition turns proliferative cells in intestinal crypts and adenomas into goblet cells. Nature 435: 959–963. 15959515 10.1038/nature03659

[33] Wong GT, Manfra D, Poulet FM, Zhang Q, Josien H, et al (2004) Chronic treatment with the gamma-secretase inhibitor LY-411,575 inhibits beta-amyloid peptide production and alters lymphopoiesis and intestinal cell differentiation. J Biol Chem 279: 12876–12882. 14709552 10.1074/jbc.M311652200

[34] Milano J, McKay J, Dagenais C, Foster-Brown L, Pognan F, et al (2004) Modulation of notch processing by gamma-secretase inhibitors causes intestinal goblet cell metaplasia and induction of genes known to specify gut secretory lineage differentiation. Toxicol Sci 82: 341–358. 15319485 10.1093/toxsci/kfh254

[35] Schroder N, Gossler A (2002) Expression of Notch pathway components in fetal and adult mouse small intestine. Gene Expr Patterns 2: 247–250. 12617809 10.1016/s1567-133x(02)00060-1

[36] Sander GR, Powell BC (2004) Expression of notch receptors and ligands in the adult gut. J Histochem Cytochem 52: 509–516. 15034002 10.1177/002215540405200409

[37] Xing F, Okuda H, Watabe M, Kobayashi A, Pai SK, et al (2011) Hypoxia-induced Jagged2 promotes breast cancer metastasis and self-renewal of cancer stem-like cells. Oncogene 30: 4075–4086. 21499308 10.1038/onc.2011.122 PMC3145824

[38] Yeo SY, Chitnis AB (2007) Jagged-mediated Notch signaling maintains proliferating neural progenitors and regulates cell diversity in the ventral spinal cord. Proc Natl Acad Sci U S A 104: 5913–5918. 17389390 10.1073/pnas.0607062104 PMC1832219

[39] Tsai S, Fero J, Bartelmez S (2000) Mouse Jagged2 is differentially expressed in hematopoietic progenitors and endothelial cells and promotes the survival and proliferation of hematopoietic progenitors by direct cell-to-cell contact. Blood 96: 950–957. 10910909

[40] Heath JK (2010) Transcriptional networks and signaling pathways that govern vertebrate intestinal development. Curr Top Dev Biol 90: 159–192. 20691849 10.1016/S0070-2153(10)90004-5

